# Differences in the diagnostic value between fiberoptic and high definition laryngoscopy for the characterisation of pharyngeal and laryngeal lesions: A multi‐observer paired analysis of videos

**DOI:** 10.1111/coa.13476

**Published:** 2019-12-06

**Authors:** Constanze Scholman, Jeroen M. Westra, Manon A. Zwakenberg, Frederik G. Dikkers, Gyorgy B. Halmos, Jan Wedman, Jan E. Wachters, Bernard F.A.M. van der Laan, Boudewijn E.C. Plaat

**Affiliations:** ^1^ Department of Otorhinolaryngology ‐ Head & Neck Surgery University of Groningen University Medical Center Groningen Groningen The Netherlands; ^2^ Department of Otorhinolaryngology Amsterdam UMC University of Amsterdam Amsterdam The Netherlands

**Keywords:** laryngeal mucosa, laryngoscopy, larynx, mucous membrane, neoplasms, pharynx, sensitivity and specificity

## Abstract

**Objectives:**

High definition laryngoscopy (HDL) could lead to better interpretation of the pharyngeal and laryngeal mucosa than regularly used fiberoptic laryngoscopy (FOL). The primary aim of this study is to quantify the diagnostic advantage of HDL over FOL in detecting mucosal anomalies in general, in differentiating malignant from benign lesions and in predicting specific histological entities. The secondary aim is to analyse image quality of both laryngoscopes.

**Design:**

Retrospective paired analysis with multiple observers evaluating endoscopic videos simulating daily clinical practice.

**Setting:**

A tertiary referral hospital.

**Participants:**

In 36 patients, both FOL and HDL videos were obtained. Six observers were provided with additional clinical information, and 36 FOL and HDL videos were evaluated in a randomised order.

**Main outcome measures:**

Sensitivity, specificity, positive and negative predictive value and diagnostic accuracy of observers using both flexible laryngoscopes were calculated for detection of mucosal lesions in general and uncovering malignant lesions. Sensitivities were calculated for prediction of specific histological entities. Image quality (scale 1‐10) was assessed for both flexible laryngoscopes.

**Results:**

HDL reached higher sensitivity compared to FOL for detection of mucosal abnormalities in general (96.0% vs 90.4%; *P* = .03), differentiating malignant from benign lesions (91.7% vs 79.8%; *P* = .03) and prediction of specific histological entities (59.7% vs 47.2%; *P* < .01). Image quality was judged better with HDL in comparison with FOL (mean: 8.4 vs 5.4, *P* < .01).

**Conclusions:**

HDL is superior to FOL in detecting mucosal anomalies in general, malignancies and specific histological entities. Image quality is considered as superior using HDL compared to FOL.

**Keypoints**

- High definition laryngoscopy (HDL) could lead to better interpretation of the pharyngeal and laryngeal mucosa than regularly used fiberoptic laryngoscopy (FOL).
- Six observers evaluated FOL and HDL videos (paired analysis) of 36 patients in a randomized order.
- Sensitivities of HDL were significantly higher compared with FOL for detecting mucosal lesions in general, differentiating malignant from benign lesions and for predicting specific histological entities.
- Although FOL is still commonly applied, it is advised to use HDL in the standard flexible endoscopic evaluation of the pharyngeal and laryngeal mucosa.

## 1 INTRODUCTION

Visualisation of the upper aerodigestive tract is essential in the diagnostic process of pharyngeal and laryngeal lesions.^1^ Especially distinguishing benign from malignant mucosal lesions is of importance because early detection of pharyngeal or laryngeal cancer improves survival.^2^, ^3^, ^4^ Although first described in 1954, flexible fiberoptic laryngoscopy (FOL) is still commonly used worldwide.^5^, ^6^ In FOL glass fibres transmit light through the fibrescope to compose the acquired image.^7^ A disadvantage of the commonly used FOL could be the overlooking of small epithelial changes. Also, the differentiation between benign and malignant tumours in vivo seems to be difficult.^4^ Very early malignant disease presents as low‐contrast unspecific mucosal changes with superficial reddening and superficial roughness.^4^, ^8^ Insufficient optical resolution negatively influences its correct interpretation, and it depends on the clinical experience whether this will be detected.^8^, ^9^, ^10^ Imaging like computed tomography, magnetic resonance imaging and fluorodeoxyglucose‐positron emission tomography provides information about the extension of tumours and lymph node metastasis but fails to identify superficial mucosal abnormalities.^10^ In the last decades, medical imaging technology has improved tremendously and this has resulted in more detailed medical photos.^11^ Next to FOL, standard definition endoscopy, also called digital chip‐on‐tip endoscopy, was introduced in 1983.^12^, ^13^, ^14^, ^15^ This endoscope consists of a small light‐sensitive charge‐coupled device (CCD) in the tip of the endoscope which functions as a miniature TV camera.^7^, ^13^, ^14^, ^15^ The image is transmitted through the endoscope to a video processor in the form of a digital signal.^15^ Image quality of distal chip technology is validated better in comparison to FOL.^16^ Nowadays, high definition images, which have 850.000 to 1 million pixels, can be used in flexible high definition laryngoscopy (HDL).^13^

HDL is a new diagnostic tool presumably leading to better interpretation of the inspected mucosa and earlier detection of head and neck cancer. However, the advantage of HDL has never been quantified before. HDL needs to pass the ubiquitous technology adoption life cycle and, like any other technology, has to be adopted by five categories of consumers.^17^ Innovators and early adopters are willing for changes and adopt new ideas but late majority need extensive statistical evidence of new technology to be convinced of the benefits for the patient.^18^ Other barriers could be the believe of medical professionals that the new technology does not show an improved performance in comparison with the standard technology.^17^

The primary aim of this study is to quantify the advantage of HDL over FOL in detecting mucosal anomalies in general, differentiating malignant from benign lesions and predicting specific histological entities in pharynx and larynx. The secondary aim is to analyse the image quality of both laryngoscopes.

## 2 MATERIALS AND METHODS

### 2.1 Ethical considerations

The Institutional Review Board of the University Medical Centre Groningen assessed this retrospective study and judged that there was no need for approval based on the Dutch Medical Research Law (Wet medisch‐wetenschappelijk onderzoek met mensen [WMO]).

### 2.2 Patients

**Figure 1:**
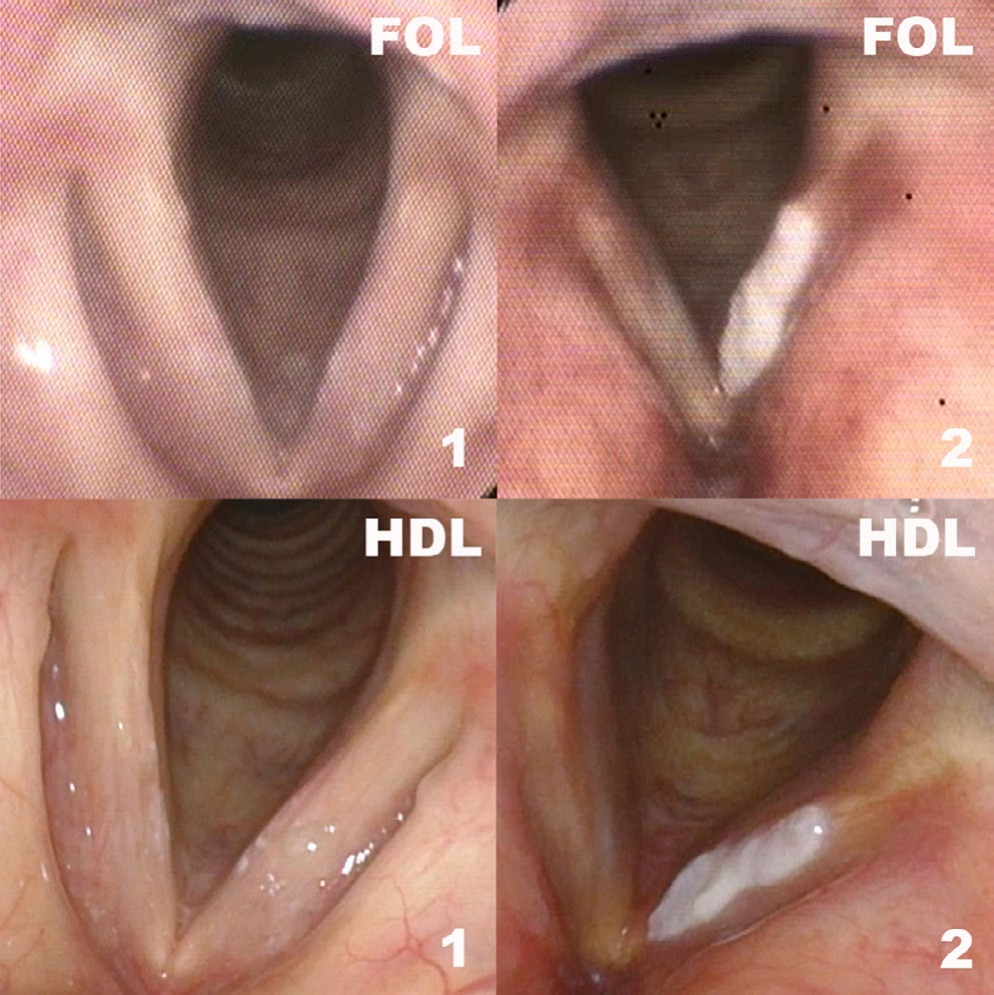
Representative pictures of FOL and HDL. FOL, fiberoptic laryngoscopy; HDL, high definition laryngoscopy; 1, normal; 2, hyperkeratosis/parakeratosis

In this study, we included archived pharyngeal and laryngeal endoscopic videos of 51 patients collected routinely during diagnostic procedures between June 2014 and October 2017. Patient data, videos and histopathological results were assembled from the electronic patient records. Inclusion criteria were availability of both one FOL video and one HDL video of the same lesion. Both videos had to be recorded within a maximum of 3 months without treatment between both endoscopies. Videos of normal pharynges and larynges were also included in order to assess the detection rate of lesions. In total, 15 lesions were excluded because either an FOL or HDL video was not available (n = 10), the lesion altered between the two video recordings (n = 1), the histological diagnosis was not possible to classify (n = 2) or the interval between the two recordings extended 3 months (n = 2). Figure 1 shows examples applied in our study group.

### 2.3 Procedure

For FOL, a flexible fiberoptic rhinolaryngoscope ENF GP (Olympus Medical Systems), which was connected to a Matrix E camera processer (Xion Gmbh), and for HDL, a flexible video rhinolaryngoscope ENF VH (Olympus Medical Systems), which was coupled to a HD monitor, were used. After using FOL for examination, patients gave informed consent and were included in a prospective HD‐NBI study. These patients (89%) underwent HDL as an additional examination for that study. In four patients (11%), FOL was followed by HDL due to uncertainty of the diagnosis. When a patient had a suspicious lesion, a biopsy was taken or the lesion was resected completely. The pathologist assigned the diagnosis of the biopsy.

The videos were edited to acquire fragments with a maximum duration of 10 seconds by using Windows Movie Maker 2012 (Microsoft Corp.). For this study, the HDL videos were edited into videos without narrow band imaging. Furthermore, we edited the videos into comparable views of the lesion, that is we showed the lesion from the same distance in both videos and gave an overview of the place of the lesion in each video. Questionnaires were made by using Microsoft Access 2010 database (Microsoft Corp.), and patient characteristics (gender, age, intoxications, brief recap of medical history) were added to each video. Questionnaires and videos were shown in random order to each observer independently by using a HD screen (Samsung Modelcode: UE50ES6100, Softwareversion: T‐MST10PDEUC‐1032.0, BT—G, SAMSUNG, Seoul, South Korea). First, observers had to classify the lesion into benign or malignant after displaying the video for maximal two times, and secondly, they had to choose a diagnosis from a presented table (Table 1). Observers had the possibility to choose from a variety of diagnoses even if there were no cases with these diagnosis included. Thirdly, observers had to judge the image quality on a scale from zero to ten (ie 0 = very poor image quality, 10 = excellent image quality). Each observer had a maximum of 30 seconds to judge each video. In this way, six observers assessed 72 videos (ie both FOL and HDL performed in 36 patients) which resulted in 432 observations. All six observers had at least 5 years of experience in the field of laryngology and/or head and neck oncology.

**Table 1:** Score list out of which observers could choose their most probable diagnosis and frequencies of FOL and HDL videos per diagnosis, respectively

Score list	Frequencies of FOL and HDL videos per diagnosis	
Diagnosis	Frequency	%
Normal	3	8.3
Cyst	0	0
Metaplasia, hyperplasia	3	8.3
Scar tissue, granulation	2	5.6
Hyperkeratosis, parakeratosis	3	8.3
Lymphoid tissue	2	5.6
Inflammation	3	8.3
Radiotherapy sequelae	3	8.3
Mild dysplasia	0	0
Severe dysplasia	0	0
Squamous cell carcinoma	14	38.9
Other^a^, *	3	8.3

Abbreviations: FOL, fiberoptic laryngoscopy; HDL, high definition laryngoscopy.

a Diagnosis papilloma, granulation or hemangioma

### 2.4 Statistical analysis

Statistical analysis was performed using SPSS version 22.0 (IBM Corp.). The sensitivity, specificity, positive predictive value (PPV), negative predictive value (NPV) and diagnostic accuracy were calculated of both FOL and HDL for the detection of a mucosal lesion and of a malignant lesion in the pharynx and larynx. Sensitivities for prediction of a specific histological entity were calculated for each laryngoscope. The chi‐squared test was used to analyse differences in sensitivities, specificity, PPV, NPV and diagnostic accuracy between both techniques. Furthermore, for each video the mean sensitivity of all six chosen diagnoses was calculated. Wilcoxon signed‐rank test for matched pair samples was performed to compare the values of FOL and HDL and to evaluate the difference in image quality between both laryngoscopes. In this study, a *P*‐value < 0.05 was considered as significant.

## 3 RESULTS

Frequencies of the included 36 specific histopathological entities are demonstrated in Table 1. Table 2 shows an overview of the patient characteristics. In total, 23 men and 13 women were included (average age 61.9 years; SD 10.47, range: 44‐84 years). The median interval between the recorded images using FOL and HDL was 1.6 weeks (SD 2.2, range: 0‐12 weeks). Of all lesions, 47.2% were located in the glottis (n = 17). The majority of the patients had a blank ENT history (63.9%, n = 23); in three patients (8.3%), a CO_2_ laser excision was performed earlier and 16.7% (n = 6) did have radiotherapy because of a laryngeal malignancy in medical history.

**Table 2:** Patient characteristics of all included patients (n = 36)

Age, years (median, range)	61.9 y; SD 10.47, range: 44‐84 y
Gender	
Men	23 (63.9%)
Woman	13 (36.1%)
Interval between recorded images (median, range)	1.6 wk; SD 2.2, range: 0‐12 wk
Location of the lesion	
Glottic	17 (47.2%)
Supraglottic	5 (13.9%)
Hypopharynx	4 (11.1%)
Oropharynx	7 (19.4%)
Hypopharynx and oropharynx	1 (2.8%)
Larynx and pharynx	1 (2.8%)
Glottic and supraglottic	1 (2.8%)

Abbreviations: SD, standard deviation; ENT, ear, nose, throat; FOL, fiberoptic laryngoscopy; HDL, high definition laryngoscopy.

### 3.1 Detection of mucosal lesions in general and differentiating malignant from benign lesions

As demonstrated in Table 3, pharyngeal and laryngeal disease was correctly distinguished from normal mucosa in 90.4% (FOL) and 96% (HDL) of 198 videos with a mucosal lesion (*P* = .03). HDLs´ specificity (50%), PPV (95.5%) and NPV (52.9%) were higher in comparison with FOL (33.3%, 93.1%, 24%, respectively, but not statistically significant (NS)). Accuracy was highest with HDL for detection of a mucosal lesion (FOL 85.6% versus (vs) HDL 92.1%, *P* = .03).

**Table 3:** Diagnostic values of FOL and HDL for detecting a mucosal lesion in general and for differentiating a malignant lesion from a benign lesion

Videos	Detection of a mucosal lesion				Detection of a malignant lesion			
	FOL (%)	HDL (%)	Χ^2^	*P*‐value*	FOL (%)	HDL (%)	Χ^2^	*P*‐value*
Sensitivity	90.40 (179 of 198)	95.96 (190 of 198)	4.81	**0.03**	79.76 (67 of 84)	91.67 (77 of 84)	4.86	**0.03**
Specificity	33.33 (6 of 18)	50.00 (9 of 18)	1.03	0.31	80.30 (106 of 132)	79.55 (105 of 132)	0.02	0.89
PPV	93.72 (179 of 191)	95.48 (190 of 199)	0.59	0.44	72.04 (67 of 90)	74.04 (77 of 104)	0.10	0.75
NPV	24.00 (6 of 25)	52.94 (9 of 17)	3.69	0.55	86.18 (106 of 123)	93.75 (105 of 112)	3.67	0.06
Accuracy	85.65 (185 of 216)	92.13 (199 of 216)	4.59	**0.03**	80.09 (173 of 216)	84.26 (182 of 216)	1.28	0.26

Abbreviations: PPV, positive predictive value; NPV, negative predictive value; FOL, fiberoptic laryngoscopy; HDL, high definition laryngoscopy.

Bold and italic values indicate the statistically significant *P* < 0.05

* Chi‐squared test.

Malignant lesions (ie squamous cell carcinoma) were correctly differentiated from both benign lesions and normal mucosa in 79.8% (FOL) and in 91.7% (HDL), that is 11.9% difference in favour of HDL (*P* = .03). Specificity and PPV did not differ between the laryngoscopes (FOL 80.3% and 72% vs HDL 79.6% and 74%, respectively, NS). NPV and accuracy were higher with HDL (FOL 86.2% and 80.1% vs HDL 93.8% and 84.3%, respectively, NS, Table 3).

### 3.2 Prediction of specific histological entities and image quality

Table 4a, demonstrates that overall sensitivity of FOL was 47.2% as compared to 59.7% of HDL (*P* < .01) in correctly predicting specific histological entities. It was found that the sensitivity for identifying squamous cell carcinoma as a specific histological entity was higher by using HDL (FOL 72.6% vs HDL 83.3%, *P* = .09) and for detecting radiotherapy sequelae with 33% higher with HDL (FOL 50.0% vs HDL 83.3%, *P* = .04). Paired analysis showed a higher sensitivity of HDL compared to FOL for mean sensitivities of predicting specific histological entities (*P* = .07) and for image quality (mean: 8.4 vs 5.4, *P* < .01, Table 4b).

**Table 4:** (a) Sensitivity per diagnosis for prediction of a specific histological entity and (b) paired analysis for overall sensitivity and image quality of FOL and HDL

(a)	FOL (%)	HDL (%)	χ^2^	*P*‐value*
Normal	33.33 (6 of 18)	38.90 (7 of 18)	0.12	0.73
Metaplasia, hyperplasia	5.56 (1 of 18)	5.56 (1 of 18)	0	1
Scar tissue, granulation	25.00 (3 of 12)	33.33 (4 of 12)	0.20	0.65
Hyperkeratosis, parakeratosis	33.33 (6 of 18)	44.44 (8 of 18)	0.47	0.49
Lymphoid tissue	25.00 (3 of 12)	33.33 (4 of 12)	0.20	0.65
Inflammation	22.22 (4 of 18)	33.33 (6 of 18)	0.55	0.46
Radiotherapy sequelae	50.00 (9 of 18)	83.33 (15 of 18)	4.50	**0.04**
Other	50.00 (9 of 18)	77.78 (14 of 18)	3.01	0.08
Squamous cell carcinoma	72.6 (61 of 84)	83.33 (70 of 84)	2.81	0.09
Overall sensitivity	47.22 (102 of 216)	59.72 (129 of 216)	6.78	***<0.01***

(b)	FOL	HDL	*Z*	*P*‐value**
Overall sensitivity (%)	47.22	59.72	2.68	**<*0.01***
Image quality (mean, min‐max, SD)	5.4, 1‐9, 1.58	8.5, 6‐10, 0.9	12.46	**<*0.01***

Abbreviations: FOL, fiberoptic laryngoscopy; HDL, high definition laryngoscopy; min, minimum; max, maximum; SD, standard deviation.

Bold and italic values indicate the statistically significant *P* < 0.05.

* Chi‐squared test, **Wilcoxon signed‐rank test.

**Figure 2:**
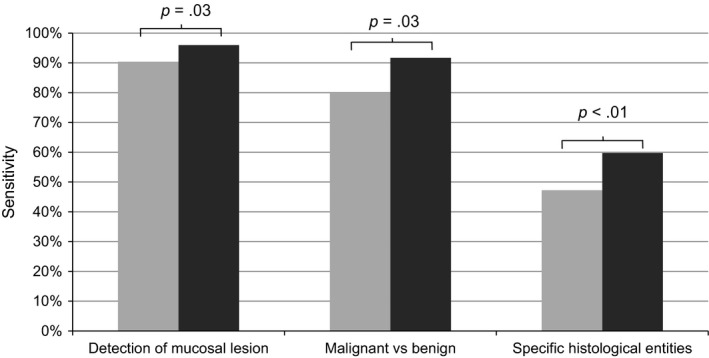
Overview of sensitivities for detection of mucosal lesions in general, for differentiating malignant from benign lesions and for prediction of specific histological entities; grey, fiberoptic laryngoscopy; black, high definition laryngoscopy

As shown in the overview of sensitivities in Figure 2, HDL reached significantly higher sensitivities compared to FOL for the detection of mucosal lesions, for differentiating malignant from benign lesions and for predicting specific histological entities.

## 4 DISCUSSION

### 4.1 Key findings

To the best of our knowledge, this is the first study using videos directly comparing the commonly used FOL with the recently developed HDL to quantify the value in the diagnostic process of pharyngeal and laryngeal lesions. This study showed that HDL is superior (*P* < .05) to FOL in the detection of mucosal abnormalities in general (FOL 90.4% vs HDL 96.0%), in discriminating malignant from benign lesions (FOL 79.8% vs HDL 91.7%) and even in prediction of specific histological entities (FOL 47.2% vs HDL 59.7%). Moreover, image quality was considered as superior using HDL compared to FOL (mean: FOL 5.4 vs HDL 8.4, *P* < .01).

### 4.2 Strengths of the study

This study was designed to combine a simulation of daily clinical practice with a reliable method of paired statistical analysis with multiple observers to obtain a scientifically solid conclusion which could be translated into daily clinical care. Daily clinical practice was simulated by using pharyngeal and laryngeal videos instead of photographs. By presenting information about the patients’ risk factors and prior medical history, it approached a routine clinical setting. The literature affirmed that motion recording of laryngeal movements has a high clinical significance, and the use of videos has numerous benefits in laryngology.^19^, ^20^ A maximum time of 30 seconds to observe a video reflects daily practice during a busy outpatient clinic.

### 4.3 Comparison with other studies

Our study shows that HDL has a significantly higher sensitivity for detecting a malignant lesion than FOL. This is of great importance because a timely detection of malignant lesions is an important factor for the prognosis of a patient.^2^, ^3^, ^4^ Additionally, early detection is essential not only in primary cases but also in the follow‐up in patients who already have head and neck cancer in their medical history.^21^ Our finding confirms the implication of the literature that FOL has difficulties in detecting minuscule epithelial and small mucosal changes and distinguishing benign from malignant tumours.^22^, ^23^ It is stated that FOL has a poor performance in recognising precancerous lesions and detection of early stage lesions even remains extremely difficult after several recurrent manoeuvres of the endoscope by skilled physicians.^23^ When we extrapolate our results, one could disclose that just by using FOL instead of HDL up to 10 out of 84 (11%) pharyngeal or laryngeal malignancies could have been missed in the outpatient setting. This could be due to the poorer image quality of FOL. Therefore, if the purpose of a flexible laryngoscopy is the detection of a malignant lesion, it is advised to use HDL instead of FOL.

The overall sensitivity to detect a specific histological entity of HDL was higher compared to FOL suggesting that HDL is a better diagnostic tool in recognising lesions. HDL also showed higher mean sensitivities of detecting specific histological entities and an enhanced image quality. These results demonstrate the advantages of endoscopes with a CCD chip. The literature shows that HDL offers optimal brightness, high quality and high resolution of the image, has a superior colour steadiness and is able to illustrate a small lesion on the mucosa clearly.^15^, ^24^ Additional imaging techniques such as narrow band imaging could even improve diagnostic accuracy and are only digitally available.^10^ Furthermore, it should be taken into account that in four patients (11%) a selection bias could have been introduced because the included lesions were difficult to diagnose. As a consequence of the uncertainty of the diagnosis using FOL, these patients underwent an additional HDL observation. This could have resulted in a lower sensitivity for both FOL and HDL. It is likely that diagnostic values would increase when lesions would be included which are more clearly to diagnose.

The overview of the sensitivities (Figure 2) shows differences in detecting mucosal lesions in general, in differentiating malignant from benign lesions and in predicting specific histological entities very clearly. Comparing these sensitivities with each other, it is obvious that they are highest for detecting mucosal lesions in general and lowest for recognition of specific histological entities. This is a logical result because it is more difficult to detect a specific histological entity than to detect an unspecific anomaly in general. Moreover, the overview explicitly illustrated that HDL has higher sensitivities than FOL.

### 4.4 Clinical applicability

Nowadays, the availability of the HDL has increased. Due to the evidence of our study and to the reflection of normal circumstances resembling daily clinical practice, our advice is to use HDL instead of FOL in the standard endoscopic evaluation of the pharynx and larynx in the outpatient setting, especially if a malignancy is expected.

## 5 CONCLUSION

HDL is superior to FOL in diagnosing pharyngeal and laryngeal lesions. Sensitivities of HDL were significantly higher compared with FOL for detecting mucosal lesions in general, differentiating malignant from benign lesions and for predicting specific histological entities. The HDL image quality was considered better. Although FOL is still commonly applied, in our opinion, it is of great importance to soon adopt HDL as the gold standard in flexible endoscopic evaluation of the pharyngeal and laryngeal mucosa.

## CONFLICT OF INTEREST

Boudewijn EC Plaat has a consultancy role for and has received research funding by Olympus Medical Systems.

## Data Availability

The datasets generated during the current study are available from the corresponding author on reasonable request.^25^

## References

[1] Paul BC , Chen S , Sridharan S , Fang Y , Amin MR , Branski RC . Diagnostic accuracy of history, laryngoscopy, and stroboscopy. Laryngoscope. 2013;123(1):215‐219. 23070976 10.1002/lary.23630

[2] Chu EA , Kim YJ . Laryngeal cancer: diagnosis and preoperative work‐up. Otolaryngol Clin North Am. 2008;41(4):673‐695. 18570953 10.1016/j.otc.2008.01.016

[3] Brouha XD , Tromp DM , de Leeuw JR , Hordijk GJ , Winnubst JA . Laryngeal cancer patients: analysis of patient delay at different tumor stages. Head Neck. 2005;27(4):289‐295. 15668927 10.1002/hed.20146

[4] Mannelli G , Cecconi L , Gallo O . Laryngeal preneoplastic lesions and cancer: challenging diagnosis. Qualitative literature review and meta‐analysis. Crit Rev Oncol Hematol. 2016;106:64‐90. 27637353 10.1016/j.critrevonc.2016.07.004

[5] Paul BC , Rafii B , Achlatis S , Amin MR , Branski RC . Morbidity and patient perception of flexible laryngoscopy. Ann Otol Rhinol Laryngol. 2012;121(11):708‐713. 23193902 10.1177/000348941212101102

[6] Hopkins HH , Kapany NS . A flexible fibrescope, using static scanning. Nature. 1954;173(4392):39–41.

[7] Winter C , Rupp S , Elter M , Munzenmayer C , Gerhauser H , Wittenberg T . Automatic adaptive enhancement for images obtained with fiberscopic endoscopes. IEEE Trans Biomed Eng. 2006;53(10):2035‐2046. 17019868 10.1109/TBME.2006.877110

[8] Gugatschka M , Kiesler K , Beham A , Rechenmacher J , Friedrich G . Hyperplastic epithelial lesions of the vocal folds: combined use of exfoliative cytology and laryngostroboscopy in differential diagnosis. Eur Arch Otorhinolaryngol. 2008;265(7):797‐801. 18057948 10.1007/s00405-007-0549-9

[9] Blitz AM , Aygun N . Radiologic evaluation of larynx cancer. Otolaryngol Clin North Am. 2008;41(4):697–713. 18570954 10.1016/j.otc.2008.01.015

[10] Zhou H , Zhang J , Guo L , Nie J , Zhu C , Ma X . The value of narrow band imaging in diagnosis of head and neck cancer: a meta‐analysis. Sci Rep. 2018;8(1):515. 29323235 10.1038/s41598-017-19069-0 PMC5765024

[11] Torkamani A , Andersen KG , Steinhubl SR , Topol EJ . High‐definition medicine. Cell. 2017;170(5):828‐843. 28841416 10.1016/j.cell.2017.08.007 PMC5714316

[12] De Groen P .History of the endoscope. Proceedings of the IEEE 2017 Oct 17:1987‐1995.

[13] Kwon RS , Adler DG , Chand B , et al. High‐resolution and high‐magnification endoscopes. Gastrointest Endosc. 2009;69(3):399‐407. 19231483 10.1016/j.gie.2008.12.049

[14] Kawaida M , Fukuda H , Kohno N . Observations of laryngeal lesions with a rhinolarynx electronic videoendoscope system and digital image processing. Ann Otol Rhinol Laryngol. 1998;107(10 Pt 1):855‐859. 9794615 10.1177/000348949810701008

[15] Kawaida M , Fukuda H , Kohno N . Clinical experience with a new type of rhino‐larynx electronic endoscope PENTAX VNL‐1530. Diagn Ther Endosc. 1994;1(1):57‐62. 18493342 10.1155/DTE.1.57 PMC2362462

[16] Plaat BE , van der Laan BF , Wedman J , Halmos GB , Dikkers FG . Distal chip versus fiberoptic laryngoscopy using endoscopic sheaths: diagnostic accuracy and image quality. Eur Arch Otorhinolaryngol. 2014;271(8):2227‐2232. 24515919 10.1007/s00405-014-2916-7

[17] Raja AS , Sullivan AF , Pallin DJ , Bohan JS , Camargo CA Jr . Adoption of video laryngoscopy in Massachusetts emergency departments. J Emerg Med. 2012;42(2):233‐237. 21215555 10.1016/j.jemermed.2010.10.020

[18] Dickson KE , Tran NT , Samuelson JL , et al. Medical male circumcision: a framework analysis of policy and program implementation in eastern and southern Africa. PLoS Med. 2011;8(11):e1001133. 22140368 10.1371/journal.pmed.1001133 PMC3226465

[19] Tsunoda A , Hatanaka A , Tsunoda R , Kishimoto S , Tsunoda K . A full digital, high definition video system (1080i) for laryngoscopy and stroboscopy. J Laryngol Otol. 2008;122(1):78‐81. 17623492 10.1017/S0022215107000072

[20] Hurford WE . The video revolution: a new view of laryngoscopy. Respir Care. 2010;55(8):1036‐1045. 20667151

[21] Nisa L , La Macchia R , Boujelbene N , Sandu K , Khanfir K , Giger R . Correlation between subjective evaluation of symptoms and objective findings in early recurrent head and neck squamous cell carcinoma. JAMA Otolaryngol Head Neck Surg. 2013;139(7):687‐693. 23788001 10.1001/jamaoto.2013.3289

[22] White JF , Knight RE . Office videofiberoptic laryngoscopy. Laryngoscope. 1984;94(9):1166‐1169. 6472012 10.1288/00005537-198409000-00007

[23] Muto M , Nakane M , Katada C , et al. Squamous cell carcinoma in situ at oropharyngeal and hypopharyngeal mucosal sites. Cancer. 2004;101(6):1375‐1381. 15368325 10.1002/cncr.20482

[24] Sato K , Umeno H , Nakashima T . Stroboscopic observation of vocal fold vibration with the videoendoscope. Ann Otol Rhinol Laryngol. 2003;112(11):965‐970. 14653366 10.1177/000348940311201109

[25] Scholman C . Differences in the diagnostic value between fiberoptic and high definition laryngoscopy for the detection of pharyngeal and laryngeal lesions: A multi‐observer paired analysis of videos. Unpublished raw data.2019 10.1111/coa.13476 PMC6972529 31747481

